# Biomaterials are the key to unlock spheroid function and therapeutic potential

**DOI:** 10.1016/j.bbiosy.2023.100080

**Published:** 2023-06-07

**Authors:** David H. Ramos-Rodriguez, J. Kent Leach

**Affiliations:** a Department of Orthopaedic Surgery, UC Davis Health, Sacramento, CA 95817, USA; b Department of Biomedical Engineering, University of California, Davis, Davis, CA 95616, USA

**Keywords:** Hydrogel, Spheroid, Adhesion, Extracellular matrix, Cell-based therapies

## Abstract

Spheroids are three-dimensional cell aggregates that mimic fundamental aspects of the native tissue microenvironment better than single cells, making them a promising platform for the study of tissue development and therapeutics. Spheroids have been investigated for decades as models in cancer research, yet we have only just scratched the surface of their potential clinical utility in cell-based therapies. Like many cells, spheroids commonly exhibit a loss of key attributes upon implantation, motivating the need for strategies to regulate their function *in situ*. Biomaterials offer numerous opportunities to preserve spheroid function and guide spheroid behavior by tailoring the local microenvironment.

## Highlights

• Spheroids are an exciting strategy to capitalize on the promise of cell-based therapies.
• Biomaterials can effectively regulate the function and behavior of spheroids *in situ* by manipulating biophysical properties such as stiffness, adhesivity, viscoelasticity, and others.
• Hydrogels and other biomaterials should be engineered with increasing complexity to mimic the local microenvironment and propel spheroids toward the clinic.

## 1 Biomaterials are essential to propel the use of spheroids in cell-based therapies

Cell-based strategies are an exciting approach for therapeutic applications in tissue regeneration and repair. Systemic or localized injection of cells results in poor survival and inconsistent behavior due to the lack of instructional cues or aberrant signaling from the diseased microenvironment. As an alternative to monodisperse cells, spheroids are three-dimensional cell aggregates that retain key aspects of the cellular microenvironment including cell-cell interactions, engagement with an endogenous cell-secreted extracellular matrix (ECM), and gradients in signaling that result in heterogeneous nutrient distribution that better recapitulate native tissues. Furthermore, spheroids secrete substantially more endogenous trophic factors that promote neovascularization and influence the inflammatory microenvironment [1], motivating their potential use as building blocks for new tissues. The formation and scalability of spheroids are far superior and more efficient compared to their more complex analog, organoids, making them an enticing candidate for large-scale manufacturing processes for clinical applications. Although cell type and spheroid size modulate spheroid behavior and dictate their therapeutic promise, the need persists for instructive cues to retain spheroid function upon implantation, avoid undesired differentiation, and reach their therapeutic potential.

Biomaterials have an essential role in the development and application of spheroid-based technologies. Beyond materials-based approaches for spheroid formation, entrapment of spheroids in tunable biomaterials has emerged as a promising strategy to instruct spheroid function and differentiation and regulate cell migration from the spheroid into the surrounding tissues. The synergistic effects of biomaterial properties and spheroid signaling, although not fully understood, directly influence cytokine production, cell spreading and migration, viability, and differentiation. Thus, intelligent selection of a biomaterial is required and should be taken into consideration to instruct spheroid behavior and achieve the desired therapeutic effect.

## 2 Biophysical properties of engineered materials regulate spheroid function

Our group has predominantly studied spheroids formed of mesenchymal stromal cells (MSCs) to potentiate their secretion of regenerative trophic factors and guide their direct contributions to tissue formation. Spheroids of other cell types are under investigation, and key factors such as cell type and spheroid diameter are intrinsically related to their desired application. Nonetheless, the interplay of additional environmental cues can affect spheroid function and instruct behavior for specific applications. Spheroid function is regulated by the biophysical properties of the spheroid carrier material or entrapment of other components within the spheroid to guide cell function and differentiation.

Spheroid function has been widely controlled by encapsulation in engineered hydrogels and controlling biophysical properties such as adhesivity, stiffness, and viscoelasticity. Alginate hydrogels covalently modified with cell-adhesive RGD (Arg-Gly-Asp) peptides are widely used as model systems and vehicles for cell transplantation. RGD-modification regulated cell adhesion, outgrowth, and tissue formation using MSC spheroids [2]. The function of stromal cell spheroids can also be dictated by controlling stiffness of the hydrogel carrier. MSC spheroids encapsulated within gelatin methacryloyl (GelMA) hydrogels of varying stiffnesses (0.5–3 kPa) exhibited proliferation, outgrowth, and differentiation changes linked to elastic modulus [3]. MSCs within spheroids in more compliant hydrogels easily remodeled their microenvironment, underwent proliferation, and migrated from the spheroid into the surrounding gel. Alternatively, spheroids in gels with high stiffnesses were exposed to a more hypoxic environment and expressed osteogenic markers. Hydrogel viscoelasticity has a profound influence on cell differentiation [4], with faster relaxing alginate gels promoting increased osteogenic differentiation. Similarly, MSC spheroid osteogenic differentiation was markedly enhanced in fast-relaxing alginate gels compared to other more elastic gels.

Instructive materials can also be incorporated within the spheroid itself to guide cell function. As spheroids initially lack an endogenous ECM, our group formed spheroids with an engineered, MSC-secreted ECM to activate integrin signaling [5]. This approach improves cell survival and increases osteogenic differentiation through α2β1 integrin binding and activation of mechanotransduction pathways (*e.g.*, Yes-associated protein, YAP). These findings demonstrate the potential of cell-based materials to increase biomineralization without the need for exogenous osteoinductive cues or growth factors. Recent advances in macromolecular crowding approaches may open the door to improve growth factor retention and influence other relevant ECM properties such as protein content or fiber alignment [6]. Carbon nanotubes, nano- and micron-sized calcium phosphates, and microparticles could also be entrapped during spheroid formation to locally deliver soluble factors in a sustained manner for improved spatial distribution of these instructive cues.

In many applications, it may be necessary to leverage biomaterials that provide structure or regulate spatial patterning to achieve desired tissue formation using spheroids. For example, silicon nanowires were applied to promote differentiation of human induced pluripotent stem cell spheroids (hiPSC) into hiPSC-derived cardiomyocytes for cardiac repair [7]. However, conductive biomaterials, such as polyvinyl alcohol and poly(3,4-ethylenedioxythiophene) (PEDOT), could yield platforms for more physiologically relevant stimulation while preserving spheroid function [8]. Bioprinting has also emerged as an exciting approach to spatially control spheroid placement and spheroid fusion in high-density engineered tissues [9]. Although hydrogels are the most common carrier to instruct cell function due to their ease of entrapping spheroids, other techniques such as electrospinning can be used to create fibrous structures. The application of versatile biomaterials that instruct spheroid function due to their morphological structure is an exciting and potentially scalable alternative for the clinical translation of spheroid-based technologies.

## 3 Engineered materials to leverage the endogenous secretome

The MSC secretome is a potent collection of bioactive factors that stimulates host cell migration and tissue repair. MSCs secrete endogenous factors such as vascular endothelial growth factor (VEGF) and prostaglandin E2 (PGE_2_) that promote angiogenesis, modulate the inflammatory microenvironment, and stimulate wound repair, and MSC spheroids secrete more trophic factors than monodisperse MSCs. MSC spheroids entrapped in fibrin hydrogels with higher elastic moduli (∼45 kPa) secreted significantly more VEGF, while PGE_2_ secretion was greater for spheroids in softer gels (∼5 kPa) [10]. To prolong the therapeutic effect of trophic factor secretion, alginate was modified with sulfate groups to locally capture and present components of the spheroid secretome to improve cell survival and resultant muscle repair [2], representing an exciting opportunity to prolong the therapeutic benefits of spheroids at the site of implantation.

## 4 The future of spheroids in regenerative medicine

Spheroids have enormous promise for use as building blocks for tissue regeneration. Although initial applications injected spheroids into diseased tissues without supportive biomaterials, substantial evidence confirms the capacity for material-driven approaches to potentiate spheroid function. However, we have yet to create materials that accurately model the complex characteristics and behavior exhibited by many human tissues. The use of biomaterials that mimic the biophysical properties of native tissues will increase the therapeutic potential of spheroids in clinical applications.

**Fig. 1:**
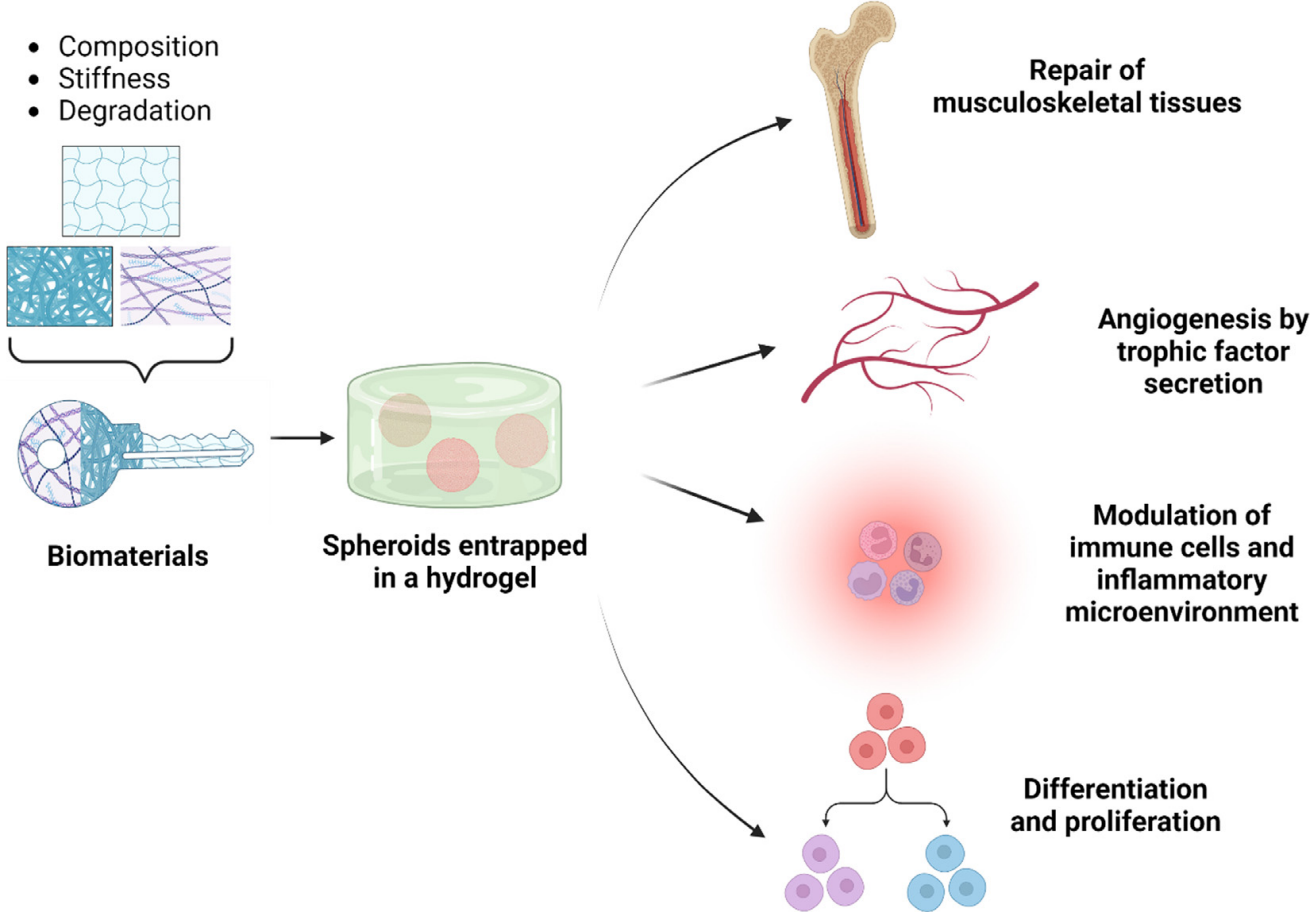
**Biomaterial selection is key to unlocking key aspects of entrapped spheroid behavior**. Biomaterial properties such as adhesive ligands, microstructure, or bioactivity are key to instructing the behavior of entrapped spheroids and unlocking their regenerative potential. Enhanced angiogenic response, augmented secretory functions to promote neovascularization or modulate the immune and inflammatory response, and regulating differentiation and proliferation are functions of spheroids with tremendous therapeutic possibilities that can be controlled *via* biomaterial interactions.

The development of biomaterials possessing gradients in stiffness, composition, and soluble cues that imitate the heterogeneity of native tissues is a step in the right direction. These attributes can be enabled by chemistries that facilitate non-invasive tunability *via* ultrasound, light, or delivery of fast-reacting small molecules that rapidly stiffen or weaken the biomaterial. Just as spheroids better recapitulate native tissues by increasing complexity from conventional 2D culture systems, we must embrace the same logic of increased complexity and multilayer design when developing biomaterials. This is made more challenging by the need to maintain biocompatibility. Nonetheless, new materials will propel the effective instruction of cell spheroids and establish them as a powerful tool for regenerative medicine, drug discovery, and disease modeling (Fig. 1).

## Declaration of Competing Interest

The authors declare the following financial interests/personal relationships which may be considered as potential competing interests:

J. Kent Leach reports financial support was provided by UC Davis Health System. David H. Ramos Rodriguez reports financial support was provided by UC Davis Health System.

## Data Availability

No data was used for the research described in the article.
